# A volumetric study of mandibular condyles in orthognathic patients by semiautomatic segmentation

**DOI:** 10.1007/s10006-021-00976-6

**Published:** 2021-06-10

**Authors:** Max-Philipp Lentzen, Maximilian Riekert, Johannes Buller, Andrea Grandoch, Matthias Zirk, Joachim E. Zoeller, Matthias Kreppel

**Affiliations:** Department for Oral and Craniomaxillofacial and Plastic Surgery, University Hospital Cologne and Faculty of Medicine, University of Cologne, Kerpener Straße 62, 50937 Cologne, Germany

**Keywords:** Orthognathic surgery, CBCT, Mandibular condyles, Segmentation, Volumetric analysis

## Abstract

**Purpose:**

This study was conducted to elucidate volumetric data of mandibular condyles of orthognathic patients by analyzing cone beam computed tomography images based upon semiautomatic segmentation.

**Methods:**

Cone beam computed tomography images of 87 patients with malocclusions were analyzed in this retrospective study. Patients were between 17 and 53 years old and diagnosed with Angle class I, II, or III malocclusion. By using the validated open-source software “ITK-SNAP,” the volumetric measurements of 174 mandibular condyles were performed. Volumetric analysis was performed according to intra-subject side differences by paired Student t test. In accordance to inter-subject side, gender, age and type of malocclusion differences bivariate analysis and ANOVA were applied.

**Results:**

The mean volume for the right condyle was 1.378 ± 0.447 cm^3^, with a maximum of 2.379 cm^3^ and a minimum of 0.121 cm^3^. The mean volume for the left side was 1.435 ± 0.474 cm^3^, with a maximum of 3.264 cm^3^ and a minimum of 0.109 cm^3^. Bivariate analysis indicated a highly significant inter-subject difference between the volume of the left and right mandibular condyles (*p* < 0.01). Females had a significantly smaller condyle volume than males (*p* < 0.05 left condyle; *p* < 0.01 right condyle).

**Conclusion:**

The fact that shape and volume of mandibular condyles show a high susceptibility to pathological alterations and particularly malocclusions makes a precise knowledge about volumetric changes indispensable. Our results show that significant inter-subject differences in condyle volume could be found with respect to the side and gender. Larger volumes could be assessed for the left condyle and for male patients.

## Introduction

The mandible takes an important part in the complex interaction of dento-maxillo-facial anatomy and physiology. Mandibular condyles, in particular, are part of growth and development affecting the dentoalveolar system [1]. Condyles offer a growth capacity, which can be triggered by intrinsic and adaptive impulses [1]. But, pathological changes can also lead to dysfunction and deformities of musculoskeletal structures and go along with temporomandibular joint disorders (TMJD) [2, 3].

Patients with malocclusion are affected predominantly and have been investigated in several studies [4–6]. Orthognathic patients with class II and III malocclusion showed major variations of the temporomandibular joint (TMJ) in size and shape [4, 7]. For instance, previous studies show that patients with a distinct overbite showed a retroposition of the mandibular condyles [8]. But besides that, also, more anterior condyle positions have been described [4]. Furthermore, dental crossbite, malpositioned, and missing posterior teeth can lead to TMJ derangements [4]. Besides the condylar position, shape and size of the mandibular condyles have been considered major factors of TMJ dysfunctions [4]. Some studies report that TMJ morphology has got a correlation with the skeletal morphology. In particular, an inverse relationship between articular eminence angle and occlusal planes is described [4]. For class III patients, a close association of condylar inclination asymmetry compared to classes I and II has been assessed. In accordance to the condylar volume, a correlation with the type of mastication has been investigated. A larger condylar width and volume was significantly associated with a hard diet. These results indicate that mastication changes can result in condylar cartilage growth and mandibular morphology [4].

An article written by Chang et al. has shown that mandibular prognathism can increase mandibular length and mandibular angles like the gonial or mandibular plane angle, as well as alter maxillofacial and morphological characteristics [9]. Facial asymmetry can be seen in patients with malocclusions and can cause significant differences between mandibular condyles [10, 11]. A relationship between condyle volume and lateral cephalogram-based registered malocclusions has been only investigated in dried Indian skulls, or by skeletal malocclusions in Japanese females [12]. Besides that, a correlation between male condylar volume and skeletal classification or a correlation between condylar volume and different parameters of cephalometric analysis has been assessed [12, 13]. Saccucci et al. compared volume and surface of mandibular condyles in a Caucasian young adult population, with different classes of malocclusion [14]. Male patients showed larger volumes than females, and significant differences in condylar volume could be shown between class II and III patients [14].

Due to that, volumetric analysis of mandibular condyles has been subject of several studies [14–18]. Computed tomography (CT) and especially cone beam computed tomography (CBCT) are part of the pre-interventional planning of orthognathic surgery patients [19]. Different studies have analyzed mandibular condyle volume by CBCT data [9, 12, 20]. But to our knowledge, a volumetric analysis by semiautomatic segmentation of CBCT datasets of patients with malocclusion has not been performed up until now. CBCT is an established technology for craniofacial imaging, has the advantage of lower radiation dose, and is applied in diagnostic of orthognathic surgery patients [21, 22]. Hence, our investigation is comprehensible and nearby.

ITK-SNAP as an open-source software provides an established and validated option for analysis of CBCT datasets [16, 23]. The software was initially used for magnetic resonance imaging (MRI) analysis of the caudate nucleus and lateral ventricle and enables the segmentation of anatomical and pathological structures in 3D datasets [24]. The applied method of active contour segmentation offers a volumetric analysis of physiological and pathological structures in 3D imaging as CT, MRI, and CBCT. The performance of CBCT datasets by using ITK-SNAP was published in several studies [25, 26]. Based on these results, several consecutive studies assessed that combining semiautomatic and manual segmentation can improve and offer precise volumetric measurement of anatomical craniofacial structures such as mandibular condyles by using CBCT datasets and ITK-SNAP [16, 27].

The aims of this study were to analyze the mandibular condyle volume in young adult subjects without TMJ dysfunction, evaluated with CBCT images, in class I, II, and III malocclusions, and to evaluate whether the condylar volume can be related to parameters like side, gender, or age.

## Materials and method

### Patients and data collection

The 3D images of 87 consecutive adult Caucasian patients (17–53 years old, 27 males and 60 females) were retrospectively analyzed and retrieved from the computer data base of our clinic for oral and maxillofacial surgery. The sample was clinically evaluated to exclude the presence of signs and symptoms of temporomandibular disorders. The patient sample consisted of three groups: Angle class I (8 patients), class II (16 patients), and class III (63 patients). All patients had undergone CBCT due to preoperative planning of orthognathic surgery between 2012 and 2017 at the clinic for Oral and Craniomaxillofacial and Plastic Surgery University of Cologne, Germany. Cone beam computerized tomography images were performed with the GALILEOS cone beam CT device (Sirona, Bensheim, Germany) at 512 pixels and a resolution of 300 mm or 2.5 line pairs/mm. The evaluation of the images was assessed by two oral radiology experts separately for each patient. Only full datasets and sufficient CBCT images were investigated and included in the study. The study protocol was approved by the corresponding medical ethical commission (approval no. 15–072). Clinical data were collected from medical records. All parameters were carefully assessed and are registered in Tables 1 and 2.

**Table 1:** Volumetric analysis according to gender in cubic centimeters

Volume	Male (n = 54)		Female (n = 120)		Total number (n = 174)	
	Left	Right	Left	Right	Left	Right
Mean	1.615	1.572	1.353	1.291	1.435	1.378
Standard deviation	0.451	0.381	0.466	0.449	0.474	0.447
Minimum	0.474	0.864	0.109	0.121	0.109	0.121
Maximum	2.327	2.379	3.264	2.104	3.264	2.379
Range	1.853	1.515	3.155	1.983	3.155	2.258

**Table 2:** Volumetric analysis according to age in cm^3^

Volume	17 ≤ 20 years		20 ≤ 24 years		24 ≤ 54 years	
	Old (n = 31)		Old (n = 30)		Old (n = 26)	
	Left	Right	Left	Right	Left	Right
Mean	1.446	1.387	1.563	1.432	1.273	1.305
Standard deviation	0.388	0.399	0.492	0.417	0.515	0.534
Minimum	0.704	0.271	0.612	0.344	0.109	0.121
Maximum	2.327	2.379	3.264	2.188	2.224	2.241
Range	1.623	2.108	2.652	1.845	2.115	2.120

Guidelines of the Declaration of Helsinki were followed.

### Statistical analysis

For the investigation of correlations of continuous variables, we applied Pearson’s test. Correlations between continuous variables and 2 categorical variables of intra- and inter-subject differences were calculated with Student t test. For inter-class differences of types of malocclusion ANOVA was performed. *P* values *p* < 0.05 were considered significant. All statistical analyses were performed using SPSS Statistics 22.0.

### Volumetric measurement

The volumetric measurement of mandibular condyles was carried out by using the open source software ITK-SNAP (Penn Image Computing and Science Laboratory) [24]. The orthognathic patients’ CBCT DICOM datasets were imported into ITK-SNAP and were investigated in sagittal, coronal, axial slices, and 3D reconstruction. The validated and previously published method of semiautomatic segmentation was used to identify and delineate the mandibular condyles [16, 27]. Semiautomatic segmentation was followed by manual segmentation to ensure correct segmentation and volumetric measurement. The volume of the 174 mandibular condyles was computed automatically in cubic centimeters by ITK-SNAP. The applied method of tissue segmentation is validated to perform morphometrical and volumetric studies based on CBCT images [25]. Tissue segmentation can be subdivided into manual, semiautomatic, and automatic segmentation [25]. The advantage of semiautomatic segmentation is the performance of efficiency and repeatability of automatic segmentation combined with the precise delineation of manual segmentation [25]. ITK-SNAP (Penn Image Computing and Science Laboratory) provides geodesic active contour and region competition methods and delivers manual and semiautomatic tools to analyze the volumes of anatomical structures such as mandibular condyles [16]. The initial validation was performed by volumetric and morphometric analysis of the caudate nucleus of the brain. Multiple consecutive studies, even on mandibular condyle measurement confirmed these results [14, 16, 27].

**Fig. 1:**
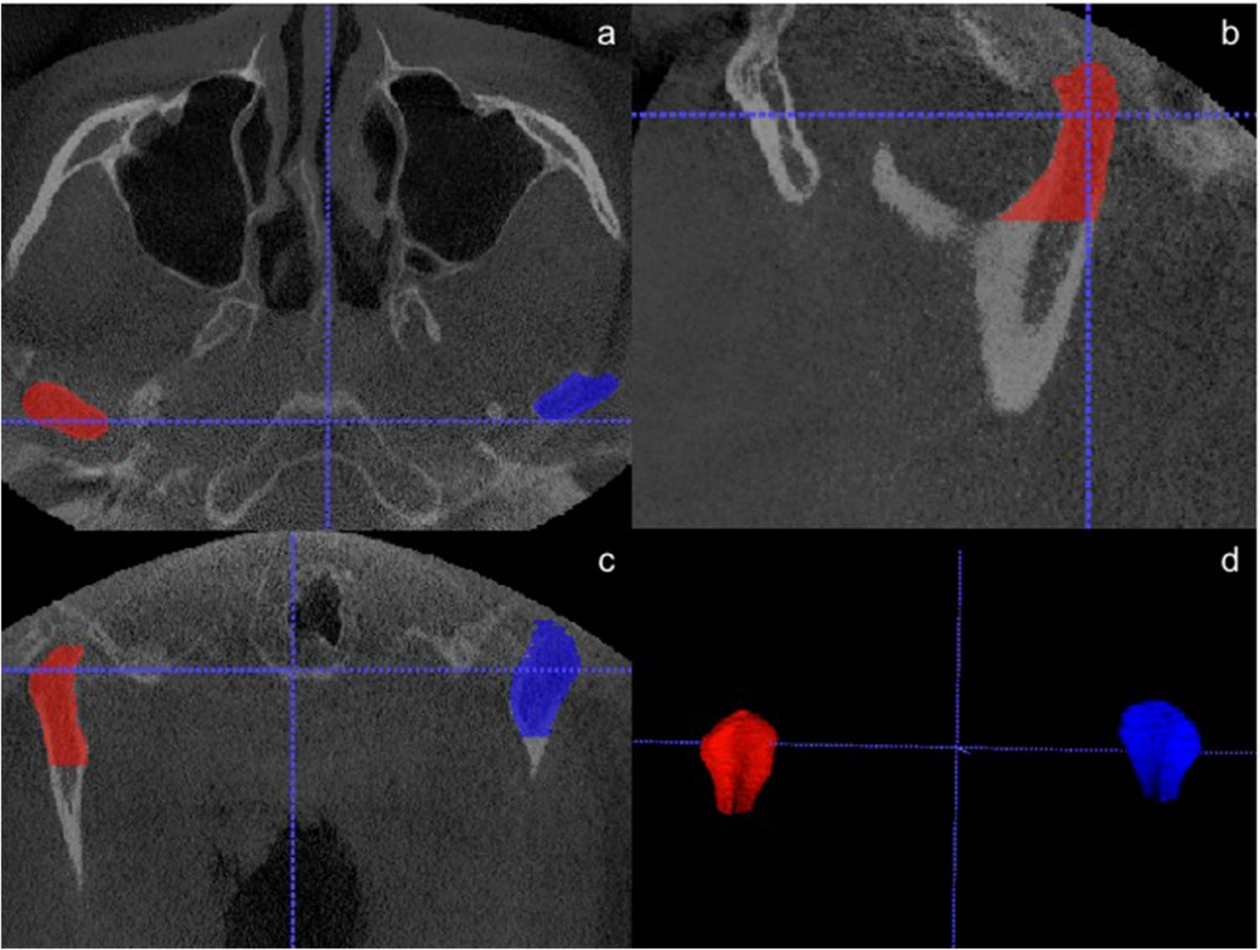
Semiautomatic segmentation of mandibular condyles by ITK-SNAP.** a** Axial plane. **b** Sagittal plane. **c** Coronal plane. **d** Three-dimensional reconstruction

Based on the measurement method by Tecco et al. (2010) and Safi et al. (2017), the superior contour was set as the upper border from the anterior, lateral, medial, and superior planes [15, 16]. Lateral borders were the most lateral extension from the sagittal, coronal, and axial view. The inferior extension was defined as the cut where the area of the coronal slices increased instead of decreasing, as the area of the beginning of the sigmoid area. Following this protocol, the condyle volume could be measured in a standardized way (Fig. 1).

## Results

This study was performed by analyzing the volume of 174 mandibular condyles of orthognathic surgery patients. The analysis investigated CBCT images of 60 female and 27 male patients. The measurements were carried out for the right and the left side, so that 120 female and 54 male condyles were assessed. At the time of diagnostic, patients had a mean age of 23 years (standard deviation 6.4 years) and a median age of 21 years. Ages ranged from 17 to 53 years.

The mean volume for the right condyle was 1.378 ± 0.447 cm^3^, with a maximum of 2.379 cm^3^ and a minimum of 0.121 cm^3^. The mean volume for the left side was 1.435 ± 0.474 cm^3^, with a maximum of 3.264 cm^3^ and a minimum of 0.109 cm^3^.

The paired *t*-test did not indicate statistically significant intra-subject differences for the right and the left condyle, regardless of gender (*p* > 0.05).

Bivariate analysis indicated a highly significant inter-subject difference between the volume of the left and right mandibular condyles (*p* < 0.01). Females had a significant smaller condyle volume than male patients (*p* < 0.05 left condyle; *p* < 0.01 right condyle). Our bivariate analysis could not indicate a statistically significant correlation between volume and age (*p* = 0.271 right condyle; *p* = 0.338 left condyle).

Furthermore, the analysis between mean condylar volumes of class I, II, and III patients in accordance to the right and left side did not indicate any statistically significant differences either (*p* = 0.098 right condyle; *p* = 0.123 left condyle).

## Discussion

The shape and volume of mandibular condyles show a high susceptibility to pathological alterations [20, 28]. These could be degenerative, caused by fractures, tumor, or inflammation and acquire accurate diagnostics and radiological imaging for a precise evaluation of the clinical situation [29]. The craniofacial growth and development of mandibular condyles predominantly affects craniofacial functions like mastication, swallowing, and speech [1]. Hence, a volumetric analysis could help to elucidate pathophysiological changes such as condyle enlargement due to anterior disc displacement, arthritis, or asymmetry of the condyles [15, 20]. Furthermore, previous investigations show a correlation of mandibular condyle volume and mandibular morphology, influenced by facial divergence and skeletal class of malocclusion [20].

The measurement of mandibular condyles of patients with malocclusion was performed by semiautomatic segmentation of CBCT images. Image segmentation can be divided into three segmentation techniques, a manual, semiautomatic, and fully automatic method [30]. The manual technique is the most user-dependent and time-consuming, but also very exact because the region of interest is outlined slice by slice [25, 30]. Fully automatic segmentation is the fastest segmentation technique, but it also causes the highest rates of inaccuracies and is therefore inappropriate for analyzing complex structures [25]. The applied semiautomatic segmentation combines advantages of efficiency and repeatability like automatic segmentation and an exact outlining of the region of interest like manual segmentation [25]. For this investigation, we performed semiautomatic segmentation by using the open-source imaging software ITK-SNAP. The program has been validated for volumetric and morphometric analysis of several anatomical and pathological structures in CBCT images of craniofacial regions, and in addition, it has also been validated for volumetric analysis of mandibular condyles [16, 31, 32]. Thus, the applied measuring method can be seen as reproducible and validated. For standardized measurements, we used the protocol published by Safi et al. and Tecco et al. before, according to the same condylar contours and extension [15, 16]. Nevertheless, our means of condyle volume are in between these studies, irrespective of the side. On the one hand, this might be caused by a young cohort between 15 and 29 years of Tecco et al., so that due to adolescence condylar volume is still smaller [15]. On the other hand, Safi et al. analyzed healthy patients where abrasions or condylar dysmorphism is not as common as in orthognathic surgery patients [16].

Furthermore, previous studies elucidated that the CBCT analysis of mandibular condyle volume of their patients ranged between 1.378 and 2.877 cm^3^ [32]. However, the investigated cohort of only 9 patients was quite small [32]. Another study reported the volume of mandibular condyles from CBCT images of 150 patients with malocclusions and assessed a mean condylar volume of 691.26 mm^3^ for male and 669.65 mm^3^ for female patients [15]. Although these studies conclude that their values should give examples of normal temporomandibular joints in the general population, their data are not in line with the values of our cohort. Safi et al. analyzed 350 patients without malocclusions and determined a mean volume of 2.278 cm^3^ for the left condyle and 2.343 cm^3^ for the right condyle [16]. Females presented a median left condyle volume of 2.126 and 2.247 cm^3^ for the right side [16]. Similar results have been elucidated by Saccucci et al., who analyzed 198 patients and assessed mean condyle volumes of 2.572 cm^3^ for the right and 2.606 cm^3^ for the left [14]. Safi et al. examined a significantly larger right condyle compared to the left condyle [16]. Further studies reported similar findings and investigated a side difference of 3.9%, whereas Safi et al. reported 6.7% [15, 16]. According to our data, we report a side difference of 4% in total, 5% for female and 6% for male patients. These numbers are in line with previously published studies [15, 16]. We assume that a general asymmetry of the human body and a preferred side for mastication could cause these results [15, 16, 33, 34]. Another investigation based on computed tomography evaluation of mandibular condyles without volumetric measurements also examined asymmetric sizes between the left and right condyles [35]. Especially, the analysis of patients with malocclusions should be performed separately for each condyle, considering the fact that in particular, these patients show asymmetrical facial structures and mastication habits.

Previously published data also show a significantly larger volume of male mandibular condyles than of female [16, 27]. These results are in line with Song et al., who found out that gender-related differences of craniofacial anatomy are common and result in female lateral facial dimensions of 97% in comparison with male lateral facial dimensions [36]. Similar results were examined by Tecco et al., who investigated a difference of 3.3% between males and females [15]. A side-dependent gender difference could be elucidated also by Safi et al.; their data report a difference between males and females of 7.7% for the left condyle and 2.5% for the right condyle and thus confirm previously published findings on sexual differences of mandibular condyle volume [16]. We also investigated gender-related differences in condyle volume. Male patients presented significantly larger condyles than females, in detail, 16% difference for the left and 18% for the right condyle, respectively.

A difference in accordance to age could not be found in the abovementioned studies [16]. But besides that, Alomar et al. assume that the mandibular condyle appearance differs greatly between different age groups, and they conclude that condyles adapt to changes of the stomatognathic system over time [1]. Considering the fact that neither previous studies nor our results examined a significant difference in accordance to age, we conclude that this could be caused by our cohort considering only patients from 17 to 54 years of age [16]. Due to our results, younger patients present larger condyle volumes than older ones, irrespective of the side. Here, the largest volumes are presented by the group between 20 and 24 years, followed by the group between 17 and 20, and finally, the smallest volumes were presented between 24 and 54 years old patients (Table 2). These results seem comprehensible, while the youngest group is still adolescent; the group between 20 and 24 years is fully grown, and the oldest group already present abrasions and atrophy in condylar volume. Nevertheless, no significant results could be presented and further investigations for proving this theory have to be made.

In accordance to patients with malocclusions, Saccucci et al. compared the volume and the shape of mandibular condyles with different skeletal pattern [14]. This study analyzed 200 patients between 15 and 30 years old and classified three groups with skeletal classes I, II, and III of patients with malocclusion [14]. They also used CBCT datasets to investigate the TMJ in accordance to volume, condylar area, and morphology [14]. Whereas this cohort could not show any difference according to the side, skeletal class III patients presented a significantly larger condyle volume compared to class I and II patients [14]. On the other hand, class II patients presented a significantly smaller condyle volume than class I and III patients, and besides that, males presented significantly larger condyles than females [14]. Thus, different classes of malocclusion appear to be associated with mandibular condylar volume and mandibular condylar area in orthognathic surgery patients. However, in accordance to different types of malocclusion, our study could not present any significant differences between class I, II, and III patients. This might be caused by a quite unbalanced distribution of the three groups of our cohort, and further investigations have to be made. Unfortunately, the study by Saccucci et al. used a different protocol than ours of volumetric measurement by using the Frankfort horizontal (FH) [14]. Due to mandibular mobility compared to FH, we assume our method to be favorable. Nevertheless, Saccucci et al. also elucidated similar gender differences and a comparable range between minimum and maximum of malocclusion patient condyle volume. Previous studies describe differences in force vectors against the condyle during mastication of patients with malocclusions. The force vector direction of class II patients seems to appear significantly larger compared to class I or III patients. Furthermore, an asymmetry of condylar inclination has been assessed in accordance to class III compared to class I and II patients [14].

Nevertheless, different parameters have to be taken into account when applying our results to clinical situations. Three-dimensional volumetric measurements are based on the correctness of segmentation. But, in some cases, an enhancement of bone voxels of the region of interest (mandibular condyle) was difficult to perform due to an insufficient suppression of the surrounding tissue. This was mainly caused by poor CBCT image quality, i.e., by patients’ movements while diagnostic was applied. Nevertheless, only datasets with adequate quality were investigated and two independent oral radiology experts performed the measurements. Besides that, our cohort consisted only of Caucasian patients who presented a severe occurrence of malocclusion so that they had to undergo surgical treatment at our clinic. Further studies have to validate our protocol with a larger, diverse cohort and restrained occurrence of malocclusion. The advantages of our study are a sufficient sample size compared to previous studies [14]. Furthermore, we used a validated method of semiautomatic segmentation based on an established protocol [15, 16]. We aimed to provide anatomical data from, to the best of our knowledge, largest study about mandibular condyle volumes of class I, II, and III malocclusion patients by semiautomatic segmentation. Furthermore, we investigated whether volumetric data correlated significantly with the clinical data of our study cohort.

From the clinical perspective, CBCT technology and 3D volumetric analysis provide further information about mandibular condyle morphology of patients with malocclusions. At most clinics, orthognathic surgery patients undergo CBCT before and after surgery. This enables the Oral and Maxillofacial Surgeon to analyze condyle morphology before and after operation and be alarmed in accordance to jeopardized patients. Our study could give additional information to assess these images and compare them with a larger cohort of patients with malocclusions. Nevertheless, with every CBCT, mainly young patients are facing a relevant dose of radiation. Thus, further CBCT imaging should be subject to patients with clinical symptoms and not be part of a regular follow-up.

A precise analysis of the volume of mandibular condyles with CBCT by semiautomatic segmentation can help to investigate pathophysiological alterations. Hence, the volumetric measurement can support structural understanding and improve individualized diagnosis and therapy of patients with malocclusion. However, further studies, especially conducted on larger cohorts, are necessary to confirm our results and to evaluate the influence of the volume of mandibular condyles in accordance to clinical symptoms.

## Conclusion

Volumetric measurement of the mandibular condyles of patients with malocclusions may serve as an important additional characteristic, derived from 3D CBCT imaging. Significant differences in volumetric measurement of mandibular condyles exist between side and gender, but not in accordance to age and difference in type of malocclusion. These results seem to be comparable to patients without malocclusions, so that condylar volume is not affected by this pathological alteration according to our cohort. But, special attention should be paid with respect to the side, gender, and patients’ age. Nevertheless, further studies, especially conducted on larger cohorts, are necessary to confirm our results and to improve the understanding of the influence of the volume of mandibular condyles on clinical symptoms of patients with malocclusions.
